# Applications of green technologies‐based approaches for food safety enhancement: A comprehensive review

**DOI:** 10.1002/fsn3.2915

**Published:** 2022-05-28

**Authors:** Fakhar Islam, Farhan Saeed, Muhammad Afzaal, Aftab Ahmad, Muzzamal Hussain, Muhammad Armghan Khalid, Shamaail A. Saewan, Ashraf O. Khashroum

**Affiliations:** ^1^ 72594 Department of Food Sciences Government College University Faisalabad Faisalabad Pakistan; ^2^ 72594 Department of Nutritional Sciences Government College University Faisalabad Faisalabad Pakistan; ^3^ Department of Food Sciences College of Agriculture University of Basrah Basrah Iraq; ^4^ 123295 Department of Plant Production and Protection Faculty of Agriculture Jerash University Jerash Jordan

**Keywords:** eco‐friendly, food safety, green technologies, microbes

## Abstract

Food is the basic necessity for life that always motivated man for its preservation and making it available for an extended period. Food scientists always tried to preserve it with minimum deterioration in quality by employing and investigating innovative preservation techniques. The food sector always remained in search of eco‐friendly and sustainable solutions to tackle food safety challenges. Green technologies (ozone, pulsed electric field, ohmic heating, photosensitization, ultraviolet radiations, high‐pressure processing, ultrasonic, nanotechnology) are in high demand owing to their eco‐friendly, rapid, efficient, and effective nature in controlling microbes with a negligible residual impact on food quality during processing. The use of green technologies would be a desirable substitute for conventionally available preservation techniques. This paper discusses different food preservation techniques with special reference to green technologies to minimize the deleterious impact on the environment and employs these innovative technologies to play role in enhancing the food safety.

## Graphical abstract

The current review article discusses different food preservation techniques with special reference to green technologies to minimize the deleterious impact on the environment and employs these innovative technologies to play role in enhancing the food safety.

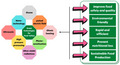

## 1 INTRODUCTION

Food is an essential part of life and human survival. Humans have been required to feed to live since the dawn of time. When the world’s population was much less, resources were plentiful, so the intention toward food processing was not quite as essential (Boye and Arcand, 2013; Wunderlich & Martinez, 2018). Barriers in food handling and storage technologies required more people to devote substantial time regularly to feed themselves and their households as the population increased (i.e., harvesting and hunting) (McClements, 2019). Food processing companies are intensively working to increase the safety of food around the world (Compton et al., 2018). There has been a significant rise in the number of incidents of foodborne illness in recent years, which has been a major public health issue (Pandiselvam et al., 2019). As a result, the food industry and customers are both concerned about microbial food safety (He & Shi, 2021). Appropriate techniques are necessary along with manufacturing and distribution systems to avoid unwanted biological and fungal spoilage, as well as to preserve the nutritional and sensory value of food (De‐Souza et al., 2018). To combat food safety issues, different strategies have been introduced such as chilling, relative humidity reduction, freezing, pasteurization, sterilization, acidification, drying, antimicrobials, and fermentation. Even so, a few of these inventions have a negative impact mostly on food’s quality, color, texture, taste, and nutrients. Furthermore, food contamination and microbial degradation are significant issues that have yet to be adequately addressed (Fung et al., 2018). Microwave and radiation frequency, cryogenic air injection, ohmic heating, irradiation, as well as other innovative food processing methods could be used to enhance the value and storage stability of different food products (Khan et al., 2017). The need for safe food manufacturing and processing methods has become much more critical as a result of population growth over the last several decades, as well as the demands arising from industrialization. Changes in the atmosphere and public health, on the other hand, have demonstrated the delicate balance between sustainable food manufacturing practices, a balanced environment, as well as a healthy society (Tekinbaş Özkaya et al., 2021). To sustain global socioeconomic sustainability, adequate supplies of safe, nutritional food would be needed. In recent times, consumers have preferred organic foods because they taste better, are additive‐free, and have a longer shelf life (Shin, 2018). Nanotechnology, ozone‐based preservation, ohmic heating, pulsed and high voltage technology, ultraviolet (UV) light technology, and radio‐frequency technology are all examples of current green technologies used in the food industry to enhance food safety. This review paper provides a comprehensive analysis of all green technologies mentioned above. The use of ozone‐based food storage technologies benefits both consumers and manufacturers. Ozone (O_3_) is an isometric type of oxygen that is formed from oxygen through lightning or ultraviolet radiation reactions and it is a strong oxidizing agent (Mohammadi et al., 2017). Along with its rapid response and good oxidative properties, ozone is now an appealing choice for both food processing and conservation industries to ensure microbial food protection. It also disintegrates rapidly into oxygen molecules (Pandiselvam et al., 2017), leaving no potentially harmful halogenated residues there in food. Furthermore, because of its high oxidation capacity (2.07 volts) in an alkaline medium, ozone is an excellent antimicrobial agent (Fisher et al., 2000). It kills a variety of microbes in small quantities and satisfies the global requirement for preservation. Furthermore, ozone therapy requires much less power unlike radiation, microwave, or heat treatment (Khadre et al., 2001). Ozone has been used for antibacterial, antiviral, antiparasitic, and also antifungal therapies for many years (Varol et al., 2017). Ozone has a strong sanitizing capability at very low concentrations. This attribute has unlocked the doors for the use of ozone to the surface applications of vegetables including fruits, sanitation of food processing devices, and wastewater processing (Qi et al., 2017). Massive developments in nanotechnology have opened up a new age of industrial technology in modern ages. The majority of nanomaterials used in goods come from many fields (Antonio et al., 2014). Textile cloth sterilization, water decontamination, medication, and food packaging have also used nanoparticles (Hajipour et al., 2013). The food sector has recently been improved by groundbreaking nanotechnology (Rossi et al., 2014). Nanoparticles with antimicrobial properties can be integrated into food packaging substances to improve shelf life and maintain food quality for consumption. Nanoparticles are anticipated to greatly increase their penetration in the food manufacturing market in upcoming years (Heinlaan et al., 2008). In the ohmic heating method, heat is used to destroy bacteria, pests, and enzymes. Thermal processing is the general name for these technologies. In the food sector, conventional thermal processing methods use high‐temperature liquid, water, or air as the heating element for the food items (Jiao et al., 2018). Due to increasing demand for high‐quality foods, green technologies stand out as a potential candidate to deliver superior and safe food products with optimum quality.

## 2 METHODOLOGY USED FOR THE CURRENT MANUSCRIPT

The current review summarizes and discusses the effect of various innovative technologies for reducing the microbial load in various foods. The effectiveness of green technologies has also been considered for food safety enhancement. Related literature was collected from 100+ papers collected using Google Scholar. Related data from different reviews and research articles were compiled. The majority of the publications are from the last decade, but some classical references have also been added.

## 3 WHAT ARE GREEN TECHNOLOGIES?

Safe or green technologies refer to the production and utilization of strategies, processes, and materials that help to save the key vital nutrients by reducing and eliminating the harmful effects involved during food processing (Iravani et al., 2017). These technologies help to prevent the food from spoilage as well as preserve the nutritional value as compared to the conventional food preservation methods. Green technology includes a broad variety of manufacturing practices that enhance food safety.

The major green technologies are discussed below;

## 4 OZONE AND FOOD SAFETY

The ozone molecules are made up of three oxygen atoms, and also their unbound electrons are arranged around an oxygen nucleus in the middle, giving it a high reactivity (Jodzis and Patkowski, 2016). Because of its rapid decomposition, ozone persists as an inconsistent gas at ambient temperature. In chilled water, ozone is much more soluble as compared to hot water, with a solubility level 13 times that of O_2_ (at 0–30°C). On account of decontamination of water, about half of the ozone is lost in 20 min. An unexpected shift in stress or temperature during electrolysis may also trigger an ozone emission (Greene et al., 2012). Ozone coalesces into a bright blue fluid at 112°C.

## 5 APPLICATION OF OZONE IN FOOD INDUSTRIES

In bacterial cytosol membrane, the cell wall is strongly and specifically oxidized by ozone. Ozone is used for disinfecting processing areas, plant machinery, and surfaces’ sterilization, and fumigation. Ozone aids in the elimination of microflora including bacteria, allowing extended food shelf life. Smith and Pillai (2004) reported that ozone showed biocidal activity against microorganisms like *Zygosaccharomyces bailii*. In vegetables and fruits processing, ozone has been utilized to eliminate pathogenic microbes, as well as mycotoxins and pesticides and chemical traces (De Souza et al., 2018). Blackberries and grapes that have been treated with ozone have a much longer shelf life and are less prone to fungal infections. The impact of ozone in its gaseous form (0–5 mg/L) or moist ozone (0–10 mg/L) was observed in carrot shelf stability. The stiffness, weight loss rate, and color of carrots subjected to gases and moist ozone were not affected (Beltrán et al., 2005). Water‐soluble ozone treatment has been added to fresh fruits and vegetables only daily to minimize microbial counts and extend shelf life. In a previous study of De Souza et al. (2018), ozone decreased the sharp rise in soluble solids throughout storage and extended the shelf life of carrots. Since the US Food and Drug Administration (FDA) authorized ozonation as a food additive, it gave a huge push to its use in multiple fruit drinks (FDA 2001). The effectiveness of ozonation for inactivating microbes from fruit juice is dependent on the pH of juices, additives (emulsifiers and sugar content), the temperature, the composition, the ozone distribution frequency, the organic substance content as well as the total solids. The impact of O_3_ on the fruit juice value is determined not only by the ozone intensity and exposure period but also by the chemical composition of juice (Choi et al., 2012). Among fruits and vegetables, stiffness is a crucial textural characteristic. Jaramillo‐Sánchez et al. (2019) explored that ozone application was used to preserve tomatoes and strawberries. Aqueous ozonation creates hydroxyl radicals throughout the media, which can open ring structures and cause formaldehyde, organic acids, including ketones to be oxidized (Patil and Bourke, 2012).

### 5.1 Poultry

Natural microflora was introduced into the broiler carcasses, which were split into breast and thigh sections. For 20 min, the inoculated culture was rinsed with 3.88 mg per liter ozone at a flow rate of 2050 ml/min. The bacterial count in the carcass was found to be lower after the carcass was washed with ozone. The shelf life of poultry meat is increased by reducing the microbial load by 2.9 log CFU Ayranci et al. (2020). The microbial numbers in the dead animal were found to be lower when the carcass was washed with ozone. Aqueous ozone treatment reduces the amount of time it takes to wash clothes and boost the shine. The cleaning cycle of dark‐fleshed fish meats was improved with ozonation (Naito and Takahara, 2006).

### 5.2 Water treatment in the food industry

Financial, ecological, and technical factors all play a role in water quality in the food manufacturing industry to meet appropriate drinking specifications. Multiple water processing techniques dependent on plasma bioreactors such as microfiltration (MF) and reverse osmosis (RO) have also been established (Noronha et al., 2002). Ozonation is gaining popularity because it has a 3000‐fold higher antibiotics’ ability than chlorine that dissolves quickly in water (Miguel et al., 2016). *Enterococcus faecalis*, *Escherichia coli*, as well as certain foodborne diseases causing microorganisms such as *Listeria monocytogenes* including *Yersinia enterocolitica*, have all been successfully removed using ozonized water (Khadre et al., 2001).

### 5.3 Pulsed electric fields and food safety

A pulsed electric field (PEF) processing method is made up of five key components: (1) an elevated power input, (2) a capacitor bank for power storage and disposal controlled by on/off button, (3) a pulse generator that uses a pulse formation mechanism (pulse‐forming network (PFN)) to generate pulses of specific voltage, design, and length, (4) a control device for regulating and controlling the parameters, and (5) treatment chambers with one or multiple electrodes (Wang et al., 2018). The fluid or aqueous material to be processed is poured into the electrode holes. Capacitors are connected in a series combination to convert 110 or 220 V into strong voltage (1100 kV). After the power storage as in a capacitor loaded with the power supply (of the opening circuit) is complete, the circuit should be closed to release the energy around the food that is to be handled (Mohamed and Eissa, 2012). Monopolar or bilateral pulses are usual modes of pulses by generating elevated PEF, and continuously decay or squared pulses are perhaps the most widely used wavelengths. Bipolar pulses are made up of one positive and one negative signal, while monopolar pulses are made up of one positive or one negative signal. A high‐frequency reversal of pulses’ polarity causes this alternating tension. A PEF process is made up of three main parts: a treatment chamber, a generator, and a controller. The PEF treatment has a wide range of applicability, encompassing fruits as well as vegetables, milk as well as meat products, and so on. Inoculating not only *E*. *coli* but also *Bacillus subtilis* throughout pea soups and treating them using PEFs of 25 to 33 kV/cm (10 to 30 cycles for 2 s) yields in 1.5D deactivation at processing temperature (Kohli and Shahi, 2017). In a persistent PEF system with only a 20 kV/cm, electric field strength, and a wide frequency range of apples, oranges, and watermelon juice, *Saccharomyces cerevisiae* was the most sensitive microorganism, accompanied by *Salmonella panama* and *E*. *coli*. Around the same treated circumstances, *L*. *monocytogenes* is perhaps the best resistant microbe. Inhibition of microorganisms requires less power at extreme temperatures. In 2 h, the baseline load is reduced from 4 log10 cycles to low or undetectable. For such a total processing time of 6 s, values in apple extracts at 24.6 kV/cm, as well as pear extracts at 22.3 kV/cm, were lowered by near to 3.15% and 38.0%, correspondingly, from their baseline values (Parniakov et al., 2015). When exposed to an external electrical domain, the electrochemical features of polarized or charged entities can cause dipolar vibration, reconfiguration, translation, and rotation. Water atoms, for example, are polarized, charged, and frequently reconstructed and reorganized as they move along the ground, all of which lead to a reduction in free energy inside the foodstuff.

Changes in molecular configuration can reduce the bioavailability of proteins, notably enzymes (Pereira et al., 2011). Hydrogen links parallel to an electrical field are intensified while those orthogonal to the ground are reduced, resulting in a reduction in the dielectric constants and a rise in water’s layer pressure. The alteration of electrically vulnerable elements with an electric field has the potential to accelerate mass and thermal transfer as shown by biological consequences that occur and which are often attributed to as (1) biomembrane permeabilization caused by elevated PEFs, (2) corona wind developed by nonuniform high voltage electrostatic fields (HVEFs), (3) heavy electric fields that catalyze molecular modification, and (4) molecules containing dipole moments that are polarized and realigned. Both these biological results are supposed to be important during the food manufacturing cycle.

### 5.4 Permeabilization effects of electric fields (EFs)

Pulsed electric field (PEF) is currently gaining popularity due to its efficient lethality against microorganisms and high extraction performance for useful components. Permeabilization of microorganisms becomes irreversible because when the electric voltage across the membranes reaches a certain threshold, it results in an intercellular substance leaking including cell lysis (Jaeger et al., 2014). The polarization impact of elevated PEF on a framework configuration on the cell surface underpins the electroporation concept. Under the influence of an electrical field, a lipid bilayer and protein of a plasma membrane become briefly vulnerable, leading to the creation of openings and an increase in cell plasma membrane permeability. Small substances like water could easily move across the plasma membranes into the cell due to cytoplasm’s colloid osmotic tension causing cell expansion, membrane breakup, and cell destruction (Golberg et al., 2010). Microbes’ inhibition is among the most important stage in the protection of value and safety features. When compared with the single conventional thermal or PEF therapy, the application of PEF and heat treatment is most successful at microbial inhibition not just for shortening treatment duration but also for significantly lowering temperature and microbial population (Bermúdez‐Aguirre et al., 2012). PEF processing (40 kV/cm, 144 vibrations) in combination with heat processing (65°C) reduced colonies by 3.6 logs in homogenized milk comprising nisin (50 IU/mL). However, the process by which PEFs inactivate spores is unknown. PEFs, according to some researchers, can cause spore germination (Shin et al., 2010).

### 5.5 Ohmic heating and food safety

Ohmic heating is among the newer manufacturing methods that have emerged in the latest 20 years. Joule warming, electrical friction heating, or electropermeable heating are all terms used to describe this method. Heat energy is produced externally and afterward transmitted to food products through conduction, convective heat transfer, and either radiation in traditional food heating systems (Varghese et al., 2014). Traditional heating procedures for items containing particle matter, particularly when the particulate matter is relatively large, necessitate such an extensive heat treatment that the particulate matter’s outer component dissolves (Knirsch et al., 2010). Food liquids including solids are warmed continuously by transmitting an electrical pulse into them in ohmic processing (clear resistance heating). Ohmic heating is identical to microwave heating, however, the frequencies are somewhat diverse (Yin et al., 2018). It not only results in the improvement of food quality but also saves money and resources for processors. Ohmic heating could produce nutritious, high‐quality foods and can be used to verify any commercial system by illustrating its use experimentally. Foods that have been ohmically processed have a life span that is equivalent to canned or sterile, aseptically manufactured foods. Electrodes that touch the food are used in most experiments, and current is transmitted through the material using several voltages including current configurations. Since the initial 1990s, the food sector has shown considerable confidence in ohmic technology, including new technologies being developed (Sastry et al., 2002). The temperature of the chilliest spot as well as the shortened residence periods invested in the warming and keeping system are the most important variables to be thoroughly measured and calculated in the continuous sterilization operation. It is crucial to assess the worst situation from a safety perspective and it is quite likely correlated with unsuspected low‐conductivity molecules in the device (Tulsiyan et al., 2008). Radio‐frequency identification (RFID) was used to assess the residing duration and residence time distribution (RTD) in solid potato grains throughout the starch solution. The impact of solid intensity and agitator spinning frequency on an RTD was investigated. The mean sample residing duration increased just like the rotary frequency increased, but there was no discernible relationship between the calculated mean specimen residing time as well as the solid concentration. The quickest particle had a frequency of 1.62 times that of the stock mean product frequency, which is crucial for food safety design methods. In situ temperature checking just like with any steady flow operation is difficult, so appropriate mathematical models and experimental validation are essential. To generate electricity, a power source (generator) is required. To push electric current across, the electrodes linked to a power source should be in direct connection with the material (Yang et al., 2014). The electrode gap (distance between electrodes throughout the system) can vary based on a system’s volume, but increasing this distance could change an electric field power, which is measured in volts per centimeter [V/cm]. Presently, ohmic heating systems are designed to work with steady process lines rather than batch lines. The substance flows in a straight line from one electrode to another, parallel to an electrical field. The electrode assembly and the pulley tube are the two key components of the collinear heater. By alternating the electrode housing/spacer tube/electrode‐housing connections, higher‐power heating systems can be achieved. Implementation voltages across electrodes as well as to the outside earthed heater covering can exceed 3–3 kV (Takhistov, 2007). As a result, all internal areas in association with food must be made of an insulating substance capable of providing long and efficient service under challenging environmental circumstances. Electrodes are positioned at different points along the material flow direction. Because of the voltage fall in the device, the product upstream has a stronger field intensity than the product downstream. Vertical to the stream direction, electrodes are mounted. The power of the electrical field remains constant across. To boost water safety, a stable ohmic heating device was designed to extract proteins from fish mince wash water obtained from the surimi processing facility (Kanjanapongkul et al., 2008). Two round 316 stainless steel metal electrodes (50 mm diameter) with such a 75 mm gap between them were mounted on each end of a cell. An ohmic cell, a conventional manual on–off adjustable transformer (0–240 V), a digital writeable power meter, as well as a mercury temperature sensor made up the constant ohmic heating mechanism and an acrylic pipe having a length of about 300 mm as well as an internal diameter around 50 mm was used to make an ohmic cell (Ayadi et al., 2004). As much as 50 to 60 Hz is the most commonly used frequency in the ohmic heating of products (Varghese et al., 2014). According to studies, electrical permeability levels for low‐frequency waves were large, and the electric permeability of turnip tissues utilized in the analysis was substantially greater for sine as well as sawtooth waves than the square wave of around 4 Hz. The heat and mass transmission characteristics of foods have been reported to be influenced by changing the frequency or wave shape of alternating current (ac) voltage throughout ohmic heating. The electrical permittivity levels, as well as the heating mechanism, are affected by the frequency, waveform of a supply voltage.

#### 5.5.1 Advantages of ohmic heating

When related to certain electroheating processes, ohmic heating has a lower initial investment. But it has a benefit across the microwave manufacturing process, where the extent to which heat can invade the food product can limit processing. Good product consistency, less processing time, lower operating costs, higher energy efficiency, and environmental friendliness are all benefits of ohmic heating (Yin et al., 2018). Previous studies have also shown that combining ohmic and plate processing will reduce the time it takes to prepare hamburger patties when compared to the traditional plate cooking method. Brunton et al. (2005) stated that ohmic heat treatment cooks meat emulsion batters quite quickly. The cooking duration was decreased by 90% to 95%, as contrasted to the conventional smokehouse cooking. A cooking thermostat of more than 75°C is needed to ensure fast cooking that satisfies minimum pasteurization requirements. The use of ohmic processing in the industry can result in energy efficiency improvements of more than 90% (Pereira et al., 2007).

#### 5.5.2 Application of ohmic heating

Electric pasteurization of milk was performed in the early 1990s by moving it in between two sheets by an electrical potential gap. Blanching, defrosting, online identification and starch gelatinization, fermentation, plucking, vaporization, drying, and extraction are some of the implementations of ohmic heating (Kumar, 2018). It has been stated that ohmic heating could be used in the processing of meat batter as well as in fish‐based items. Multiple studies have looked at a variety of perspectives of ohmic heating’s use in the food sector, such as its ability to improve dye diffusion within beet. The impact of ohmic cooking on the lipase function was investigated, as well as the impact of ohmic cooking on phytochemicals as the antioxidant function of rice bran collected from various ohmic heating circumstances (Loypimai et al., 2009). Aseptic manufacturing has been commonly employed in the food sector for pasteurization and sterilization of fluid foods like milk including fruit juices across the last few decades. Because of these advantages, immediate heating via a Joule effect, like ohmic heating, has achieved popularity in the food sector. It is due to the likelihood of using a constant aseptic treatment to treat particulate foods. Following future legislative authorization, an ohmic heating system has the potential to be one of the army ration programs’ advanced technology implantations for enhancing storage life and quality improvement of both individuals’ and teams’ feed feeding applications (Goullieux and Pain, 2014).

## 6 PHOTOSENSITIZATION AND FOOD SAFETY

Photosensitization, also recognized as photodynamic treatment, has recently sparked concern in food science, with positive outcomes throughout food systems such as substantial inhibition of planktonic bacteria as well as *Vibrio parahaemolyticus* biofilms, and successful disinfection of beef, pork chops, poultry, and apple pretreated both with *Staphylococcus aureus*, *E*. *coli* (Seidi Damyeh et al., 2020). A cytotoxic singlet oxygen (1O_2_) is generated by an easy and manageable photosensitization mechanism from three nonpoisonous components: photosensitizer, illumination, and oxygen. Free radical is generated from activated photosensitizers by moving electrons in oxygen and then reducing the resulting reactive oxygen molecules, for example, hydroxyl radicals as well as superoxide anion (DeRosa and Crutchley, 2002). As a result, several cytotoxic molecules would be created, resulting in DNA, plasma membrane, and enzymes’ destruction. The efficacy of photosensitization as an antimicrobial strategy against a broad variety of microorganisms, like parasitic protozoa, mammals’ viruses, and bacteriophages, fungus, Gram‐positive bacteria as well as Gram‐negative in vegetative type spores, or biofilms, is one of the most significant features of the treatment (D’Souza et al., 2015). This innovation has lately been verified as a feasible antimicrobial strategy in food‐related uses, with wide‐spectrum effectiveness against bacteria like *Listeria monocytogenes*. Curcumin has been shown to display photoinactivation of such well‐structured populations of cells, resulting in a 4.9 log CFU (colony‐forming units’) decline in *Listeria innocua* microorganism (Bonifácio et al., 2018). The efficacy of this therapy can be influenced by several factors, including curcumin intensity and light dose. The concentration dependence of photoinactivation employing curcumin has also been stated in one of the reviewed articles (Bhavya and Hebbar, 2019). Curcumin (1.60 mM) caused an 8 log CFU/mL decline in *E. coli*, while 20 mM curcumin caused 5.94 and 5.91 log CFU/mL reductions in *E. coli* and *S. aureus*, respectively (Bhavya and Hebbar, 2019). Curcumin has been encapsulated to increase its photoactivity. By coating curcumin with silica nanomaterials, researchers were able to demonstrate an improvement in cellular uptake or phototoxicity antagonistic toward oral carcinoma cells as well as tumor spheroids (Table 1, 2, 3).

**TABLE 1:** Applications of ohmic heating in food processing and preservation

Applications	Advantages	Food items	References
Sterilization, heating liquid foods containing large particulates and heat‐sensitive liquids, aseptic processing	Attractive appearance, firmness properties, pasteurization of milk without protein denaturation	Cauliflower florets, soups, stews, fruit slices in syrups and sauces, ready‐to‐cook meals containing particulates, milk, juices, fruit purees	(Sandrine et al.,^2001^) (Pataro et al., 2011)
Ohmic cooking of solid foods	The cooking time could be reduced significantly. The center temperature rises much faster than in conventional heating, improving the final sterility of the product, less power consumption, and safer product	Hamburger patties, meat patties, minced beef, vegetable pieces, chicken, pork cuts	(Bozkurt et al., 2009) (Zell et al., 2009)
Space food and military ration	Food reheating and waste sterilization. Less energy consumption for heating food to serving temperature, products in reusable pouches with long shelf life. Additive‐free foods with good keeping quality of 3 years.	Stew‐type foods	(Jun et al., 2007) (Yang et al., 2002)
Ohmic thawing	Thawing without increase in moisture content of the product	Shrimp blocks	(Roberts et al., 1996)
Inactivation of spores and enzymes	To improve food safety and enhance shelf life, increased stability and energy efficiency, reduced time for inactivation of lipoxygenase and polyphenol oxidase, inactivation of enzymes without affecting flavor	Process fish cake, orange juice, juices	(Loypimai et al., 2009)
Blanching and extraction	Enhanced moisture loss and increase in juice yield	Potato slices, vegetable purees Extraction of sucrose from sugar beets, extraction of soymilk from soybeans	(Wang and Sastry, 2000)

**TABLE 2:** Bacterial inactivation reported in different foods by using high‐pressure processing (HPP) treatment

Technique	Food	Reduction of strains	References
HPP	Chopped raw meat	It has been observed that *E. coli* O157:H7 reduced from 5.9 colony‐forming units (CFU) to 5 CFU after treatment at 700 MPa. 1 min, 15°C	Gola et al., 2000
HPP	Apple (pH 3.5)	*E. coli* O157:H7 has been reduced up to >5 logs by applying 500 MPa at 25°C and for 5 min	Jordan et al., 2001
HPP	Milk	*Bacillus* spores are reduced up to 5.9 logs by applying 800 MPa at 40°C for 5 min and nisin (104 IU/mL) with double cycling	Black et al., 2008
HPP	Cheese	2.4 log reduction with nisin with 60 MPa for 210 min at 30°C (germination step) + 400 MPa for 30 min at 30°C *Bacillus* spores	López‐Pedemonte et al., 2003

**TABLE 3:** Bacterial inactivation has been detected in different foods after treatment with pulsed electric field (PEF)

Name of the technique	Food	Reduction of strains	References
PEF	Milk	When examined independently, *lactic acid* bacteria and entire *Staphylococci* had 3.9 ± 0.2 and <1.5 log colony‐forming units (CFU)/ml, correspondingly	Pescuma et al., 2010
PEF	Meat	*Bacillus cereus* was found in 350 samples of commercial meat foodstuff in Brisbane, with just an average of 3.4 log CFU/g	Eglezos, 2010
PEF	Rice	According to risk evaluation tests performed in Shanghai, China, 70 samples of cooked rice were taken, where one of them showed ~104 CFU/g *Bacillus cereus* and the remaining samples had *B*. *cereus* just below the permissible limit	Dong, 2013
PEF	Pasta	*Bacillus cereus* (~109 CFU/g) has been identified as causative organism of food poisoning that has resulted in death. The dish was consumed 5 days after it was prepared	Naranjo et al., 2011

## 7 ULTRAVIOLET (UV) LIGHT AND FOOD SAFETY

The microbicidal action of UV light that generally ranges from 200 to 280 nm is fully identified. UV light is mainly used in food safety to pasteurize liquid food products and beverages. The key benefit of UV innovation over traditional heat processing is that it produces higher‐quality food without jeopardizing its safety (Ramesh et al., 2016). UV has proven to be among the most powerful methods for killing microorganisms while causing limited depletion of nutrients. UV satisfies the market demand for extremely sustainable, additive‐free, clean, and wholesome foods in this aspect (Falguera et al., 2011). TiO_2_, also identified as titanium oxide, seems to be a nontoxic, nonhazardous inactive substance. TiO_2_ has also been commonly used to eliminate airborne bacteria, to combat ambient air pollution for the treatment of cancer, to shield plants against disease, to purify water, to clean drinking water, as well as to sterilize food packaging content and surfaces. It is utilized as a coloring agent throughout the food processing industry, specifically for confectionery, clear sauces and bandages, and even certain powdered foods (Ammendolia et al., 2014). Microbes, such as bacteria, fungus, alga, and viruses, are inactivated or destroyed by TiO_2_ photocatalysts. Reactive oxygen species (ROS) may cause chemical alteration or cleavage of genetic material and cell membranes, disrupting and damaging cell processes and structures (Kim et al., 2013). Furthermore, when the bacterial cell was subjected to UV‐assisted TiO_2_ photocatalysis (TUV) treatment, the authors reported that significant cell damage (devastation of the plasma membranes, DNA, and internal organelles) resulted. It was accompanied by an elevated cell wall disruption, which leads to ion release, and that this destruction would be permanent, resulting in cell mortality (Hitkova et al., 2012). TUV innovation has proven to be much more effective than UV in disinfecting drinking water as well as sewage due to its strong photocatalytic activity. By destroying algal blooms and sewage, groundwater, TUV has raised interest in algae growth management and sewage and groundwater management. TUV treatment now for food safety, on the other hand, is gaining popularity (Ramesh et al., 2016).

## 8 HIGH‐PRESSURE PROCESSING AND FOOD SAFETY

High‐pressure processing (HPP) has recently been the subject of research into how it induces bacterial, viral, as well as fungal elimination and enhances food safety. As a consequence, there has been considerably increased interest in studying the mechanism to stress generated by the microbes of focus at the genomic as well as proteomic levels (Palou et al., 2002). HPP was found to not only preserve the sensitive sensory characteristics of avocado but also to ensure a generally safe and sustainable storability. Microarray, as well as proteomic studies, has provided useful information on potential genes or enzymes associated with HPP susceptibility or preservation. Food microbes like *E*. *coli* as well as *L. monocytogenes* have been the focus of this study (Considine et al., 2008). HPP does have the ability to generate high‐quality foodstuffs with fresh product features that are microbially safe, and also have a long shelf life. HPP foodstuffs are now classified under novel foods because they meet two factors: they were created using a novel processing technology and they have a background of human utilization (Shipton et al., 2005). The expansion of life span while keeping the sensory features of products is among the key objectives of HPP (Patterson et al., 2005). Moreover, relying on the processing circumstances (temperature, duration, and pressure), as well as the variety of proteins, HPP‐induced enzyme gelatinization can be progressive. Proteins frequently, but not always, degrade, dissolve, or precipitate reversibly under HPP values of 100–300 MPa (Rastogi et al., 2007). The cellular morphology and essential processes of the bacterial cells are significantly affected by high‐pressure treatments. HPP’s deadly influence on microbial populations is thought to be caused by a combination of impacts on cell membrane conductivity, cell shape, changed metabolic responses, and interference with the heredity mechanism that happens in microorganism cells (Daher et al., 2017; Barák and Muchová, 2013). Peptidoglycans make up the bacterial cell wall (that consists of N‐acetylglucosamine and N‐acetylmuramic acid, and three amino acids, i.e., D‐glutamic acid, D‐alanine, and meso‐diaminopimelic acid). The bacterial cell membranes would be a bilayer of proteins as well as phospholipids that distinguish the inside cells from their surrounding environment (Lee et al., 2020). High‐pressure treatments cause the bacterial plasma membrane to be disrupted, resulting in enhanced permeability as well as increase in food safety. In phosphate‐buffered and tuna flesh slurry, HPP was shown to be powerful at killing histamine‐producing bacteria. *Photobacterium phosphoreum* was shown to be quite resistant to the other bacteria examined, and the treatment caused substantial cellular membranes as well as cell wall degradation, as observed by scanning electron microscopy (*SEM*). It was confirmed that gestation of HPP‐handled cells on the magnesium‐rich media had stabilizing influence on ribosome composition, and so the deterioration of cell viability could have been attributed to a deficit of metallic ion solutes even after cell deterioration, as well as ribosome composition’s conformational modifications detected not only during pressure‐induced (500 MPa/30 min/25°C) *E*. *coli*, *Salmonella*, but also *S. aureus* destruction in milk owing to morphological changes including peptidoglycan layer disintegration which prevent from spoilage and enhance food safety (Yang et al., 2012). A single HPP process has three stages: pressurization, pressure‐hold, and depressurization, every each one of which has a varying effect on bacterial activity. A conventional kinetic model was used to explain the inactivation rates inside the dynamic phase in respect of pulse effect levels and the isochoric holding phase (Woldemariam and Emire, 2019). The effects of pressurization and depressurization were also cumulative. Like a new and revolutionary technology that could meet the same food safety criterion as heat pasteurization while also addressing the demand toward fresh‐tasting minutely treated foods, HPP has enormous potential and demonstrated success within the food sector (Morgan et al., 2000). HPP may inactivate bacteria and enzymes, as well as change structure, having a minimum or no impact on food nutritive and sensory qualities. HPP has also been demonstrated to operate synergistically with certain other bacterial agents like lacticin 3147, inositol, and nisin to improve bactericidal actions and food safety. Several pressure‐transmitting media (PTMs), including distilled water, glycol–water solution, and castor oil, are used to transfer pressure inside a vessel. The pressure‐transmitting medium (PTM) is determined based upon absorption coefficient and effort to prevent vessel degradation. The amount of distilled water might decrease by up to 15% (around 600 mega pascals (MPa)) at 2–3°Celsius based upon the pressure employed (per 100 mega pascals) and temperatures have risen, according to reports by Balakrishna et al. (2020). Glycol (25%–75%) and 2% benzoic acid were employed as PTMs in an attractive investigation to investigate the perceived rise in pressure and deactivation of *Bacillus subtilis*. The initial pressurization (759 MPa/10 min) was performed at 30°C and the temperature change was detected following pressurization Sehrawat et al. (2021). Since it was based on fluid characteristics, the rise of temperature from over the starting point temperature was 22°C as in the case of 2% sodium benzoate (min) and 26–27°C in 75% glycol comprising 25% water. When benzoic acid solution was employed as a PTM, the deactivation of *Bacillus subtilis* spores was maximized.

## 9 ULTRASOUND AND FOOD SAFETY

Ultrasound treatment has been extensively used for the preservation and valorization of nutrients in food resources with a high efficiency (Li, Zhang, et al., 2021). By combining ultrasonic and sanitizer applications, the number of *Escherichia coli* O157 spot‐inoculated onto the top portion (flat layer) of spinach was reduced. Medicines with ingredients such as ASC (200 milligrams per liter) for 2 min decreased *E. coli* number around 2.1 logs above water wash, even though other disinfectants only decreased *E. coli* number by around 1 log cycle. When ultrasonic was administered to a production wash, several earlier research found that it was less successful at reducing wild flora as well as human pathogen concentrations from product surfaces (Olaimat and Holley, 2012). When it comes to using ultrasonic in a washing operation, there may be a certain thing to keep in mind. Soluble gas in even a soap solution, for example, is known to reduce cavitation action in a wiping process. As a result, any ultrasonic washing application requires degassing. Furthermore, according to such a standing wave development, the acoustic site spread inside an ultrasonic therapy container or tank is not consistent (Zhou et al., 2009). Variations will occur due to the nonuniform ultrasonic current distribution as well as nonuniform decomposition in microorganism inactivation activity in various parts of a washing container. As a result of the uneven sonic field allocation, those that generally tend to leave those that have obtained a better dose of ultrasound treatment and therefore have a minimal microbial count, which could be smoothly passed by neighboring leaves that have got less ultrasonic treatment and therefore have elevated microbial activity during rinsing therapy. Ultrasound has been utilized in food processing for physical and chemical modification of natural compounds (Li, Zhang, et al., 2021). A comprehensive understanding of both the underlying principles of powerful ultrasonics and a competent layout of the washing system and operating process is required to completely release the capability of ultrasonics in product disinfection applications.

### 9.1 Nanotechnology and food safety

Another possible application of nanotechnology is now the identification of minute quantities of a chemical pollutant, virus, or bacteria within the food chain. These nanomaterials can then target any food microbe with precision. Cornell University has already explored the use of nanotechnologies to identify pathogenic species in food as well as the establishment of nanofood preservation (Alfadul and Elneshwy, 2010). Nanotechnology has designed a *Staphylococcus aureus* identification test dependent on magnetic particles that can detect the majority of *S. aureus* species at a quite small viable count. As a consequence, food safety would be improved.

## 10 BIOPOLYMERIC NANOPARTICLES

When food is not going to be eaten right away after it has been made, to protect the foods from debris, air, sunlight, microbial pathogens, humidity, and several other damaging or toxic materials, the wrapping should also be secure under its desired standards of use, harmless, inexpensive to manufacture, lightweight, simple to discard of or replace, able to endure severe processing or packing conditions, and impermeable to some environmental factors (Duncan, 2011). Polylactic acids (PLAs) are of the very important components in environment‐friendly biopolymeric nanomaterials. PLA is commonly accessible from a variety of companies and is frequently utilized to encapsulate and also to distribute medications, vaccines, and enzymes as well as to enhance food safety, but it does have some drawbacks: It is easily eliminated from the circulatory system and remains mostly in the liver and kidneys from where it is segregated (Ravichandran, 2010).

## 11 NANO‐ENCAPSULATION

Through nano‐encapsulation, food ingredients are encased in a nanostructured polymer. Polylysines preserve oil from oxidation by acting as an antioxidant. Encapsulation with curcumin of hydrophobically adapted starch increased its anticancer ability (Sekhon, 2010). Using nanoparticles formed from montmorillonites, silver zeolite, safe‐to‐eat nanoparticle coatings based on chitosan were developed. Silver and silica are examples of inorganic nanosized materials and coatings that have been utilized as preservatives and additives that enhance the taste and spice as well as food safety.

## 12 NANOLAMINATES

Another nanoparticle strategy is commercially feasible for the food sector, in addition to nanodispersions as well as nanocapsules. The nanolaminate is an exceedingly fine food‐grade coating (1–100 nanometers) with physically linked or covalently linked dimensions composed of two and sometimes more nanometer‐sized films of material. While polysaccharide and enzyme coatings are effective at blocking oxygen as well as carbon dioxide, they are ineffective at keeping moisture out. The nanolaminates, which are based on lipids, are effective in shielding the food from humidity and by doing this they prevent the spoilage of food (Morillon et al., 2002).

## 13 SILVER ZEOLITE

Silver zeolite is produced by mixing alkaline earth metals with crystals of alumino‐silicate, which is then partially substituted by silver ions utilizing the ion exchange process. Antibacterial silver zeolite encased ceramics are being used in a range of items including food protection and shelf life‐extension, medical device disinfection, and sterilization. Silver‐based nanoparticles demonstrated persistent antimicrobial efficacy in contrast to zeolite‐based substances, making them ideally suitable for long‐term food wrapping (Inoue et al., 2002).

## 14 NANO‐ALUMINO‐SILICATE

The application of alumino‐silicate nanocomposites with active additives is one similar attempt. Alumino‐silicate nanoparticles dispersed on crop surfaces are quickly picked up by insect feathers, which is an attribute. This is a biologically effective and environment‐friendly pesticide (Sharon et al., 2010). At the nanoscale, prominent chemical companies are developing effective pesticides. The usage of alumino‐silicate is such a notable endeavor that plays an important role in food safety by killing insects. One such effort involves the use of alumino‐silicate nanotubes containing active compounds. Alumino‐silicate nanostructures have the benefit of being lightweight. Insect hairs can take up chemicals sprayed on various substrates. Insects groom themselves regularly and ingest pesticide‐laced nanotubes. They are indeed biologically quite active and, in general, more active insecticides that are not harmful to the environment, so these technologies are known as eco‐friendly.

## 15 CONCLUSION

Green technologies aim to produce food that is both safe and of high quality. Replacement techniques could improve organoleptic and sensory properties while also ensuring food microbial protection. PEF, UV, nanotechnology, ohmic heating, and ozone are examples of novel food manufacturing technologies that have decreased energy usage, emissions’ reductions, increased efficiency, enhanced productivity, and enhanced product quality. Combining these strategies could save resources while also improving food safety and food quality. The use of all these green techniques in the food sector has been investigated. However, more research is needed to determine the best treatments to combine to minimize the intensity of single‐system processes and increase the overall characteristics of food and the safety of food.

## CONFLICT OF INTEREST

The authors have declared no conflict of interest.
